# Comparison of Static Balance Control in Infected Htlv-1 Subjects with Different Tsp/Ham Diagnosis

**DOI:** 10.3390/v14112334

**Published:** 2022-10-25

**Authors:** Kelly Helorany Alves Costa, Patrícia Seixas Alves Santos, Gizele Cristina da Silva Almeida, Andrew Sousa Caires, Beatriz Helena Baldez Vasconcelos, Ramon Costa Lima, Mariangela Moreno Domingues, Maria da Conceição Nascimento Pinheiro, Rita Catarina Medeiros Sousa, Anselmo de Athayde Costa e Silva, Givago Silva Souza, Bianca Callegari

**Affiliations:** 1 Núcleo de Medicina Tropical, Universidade Federal do Pará, Belém 66005240, Brazil; 2 Instituto de Ciências Biológicas, Universidade Federal do Pará, Belém 66075110, Brazil; 3 Universidade Federal de Rorraima, Boa Vista 69310000, Brazil; 4 Instituto de Ciências da Educação, Universidade Federal do Pará, Belém 66075110, Brazil; 5 Instituto de Ciências da Saúde, Universidade Federal do Pará, Belém 66005240, Brazil

**Keywords:** HTLV-1, tropical spastic paraparesis, postural balance, stabilometry

## Abstract

(1) Background: Tropical spastic paraparesis (TSP/HAM) associated with the T cell lymphotropic virus in type I humans (HTLV-1) is a slow, chronic, and progressive disease that causes balance changes. TSP/HAM diagnosis can be classified as probable, possible, and definite. We compared the static balance control of HTLV-1-infected patients with different TSP/HAM diagnosis. (2) Methods: Our sample consisted of 13 participants infected with HTLV-1 and 16 healthy participants. The center of pressure was recorded using a force platform with open and closed eyes. We divided the recordings into three intervals, period T1 (corresponds to the first 10 s); period T2 (from 10 to 45 s); period T3 (from 45 to 55 s). (3) Results: Eight participants infected with HTLV-1 were classified as probable TSP/HAM and five participants infected with HTLV-1 were classified as definite TSP/HAM. There was a significant increase in postural instability in patients with definite PET/MAH considering the structural and global variables of body sway compared to the control and the probable TSP/HAM. (4) Conclusions: We concluded that the severity of balance is directly related to the degree of signs and symptoms of TSP/HAM.

## 1. Introduction

About 15 and 20 million people worldwide are infected with the human T cell leukemia virus type 1, also known as HTLV-1 [1]. Viral infection occurs during sexual intercourse [2,3], blood transfusion [4], or through vertical transmission from mother to child [5,6,7].

HTLV-1-infected subjects can lifelong remain without clinical complaints. However, some individuals (2–5%) can experience adult T cell leukemia or HTLV-1-associated myelopathy/tropical spastic paraparesis (TSP/HAM) [8,9,10]. TSP/HAM is a chronic presentation with a slow progression that mainly affects the spinal cord [8,11,12].

TSP/HAM is characterized by a degeneration of the white and gray matter of the spinal cord due to a chronic mononuclear inflammatory infiltrate [13]. The evolution of TSP/HAM is represented by a loss of motor and sensory neurons in the anterior and posterior spinal cord columns [13]. Progressive gait deficits with spastic paraparesis, hyperreflexia, clonus, and autonomic dysfunction are commonly found in patients with TSP/HAM [9].

In 1988, the World Health Organization elaborated a diagnostic guideline for TSP/HAM that was reviewed later by other authors [12,14]. It was proposed to stratify the clinical presentation of TSP/HAM at different levels, called definite and probable TSP/HAM [12,15]. Definite TSP/HAM was the simultaneous presentation of chronic progressive symmetric spastic paraparesis of HTLV-1 antibodies in cerebrospinal fluid (CSF) and serum, while probable TSP/HAM was progressive myelopathy and had the presence of HTLV-1 antibodies in serum or CSF, but not both. Additionally, it was suggested that a model with a third level of clinical presentation was named as possible TSP/HAM, which was an incomplete clinical presentation, and the presence of antibodies in the serum and or CSF was confirmed by a positive polymerase chain reaction (PCR) for HTLV-1 in blood or CSF [14].

Many investigations reported a loss of static balance control in patients infected with HTLV-1 [16,17,18,19,20,21,22,23] using a different method for assessment, such as the Berg scale [18], Tinetti test [16,24], force platforms [23], and baropodometric platforms [18,19,20,22]. Most of these investigations have indicated that carriers of HTLV-1 without clinical symptoms can also present mild balance-control losses compared to the severe losses that are possible in patients with TSP/HAM [22,23].

The interpretation of the static balance evaluation using a force platform is usually based on the analysis of approximately 60 s of body movement recordings [25]. However, variations in the magnitude or frequency of body sway probably occur during the time course of these recordings. It was identified that short- and long-term mechanisms are involved in postural control during quiet standing [26].

In the present study, our objective was to compare static balance control during different time-series periods of the center of pressure (COP) sway from the controls and participants with HTLV-1 with varying levels of TSP/HAM diagnosis, as indicated by previous studies [12,14]. We hypothesized that especially subjects with a definite diagnosis of TSP/HAM had a loss of balance control throughout the time course of the stabilometric examination.

## 2. Materials and Methods

### 2.1. Ethical Considerations

The local Ethics Committee for Human Research approved all the procedures of the present investigation (Nucleo de Medicina Tropical, Universidade Federal do Pará, Brazil, report #633.187) that agreed with the Declaration of Helsinki. All participants were informed about the experimental procedures and gave their written consent to participate in the experiments.

### 2.2. Subjects

The present investigation recruited 29 subjects (13 participants infected with HTLV-1 and 16 HTLV-1-non-infected participants). HTLV-1-non-infected subjects (control group) were healthy and had no history of chronic degenerative diseases, while subjects infected with HTLV-1 (HTLV-1 group) had a diagnosis confirmed by a polymerase chain reaction (PCR). A neurologist evaluated all participants infected with HTLV-1, following the criteria for the classification of TSP/HAM [14], to identify the level of determination of TSP/HAM. After a neurological evaluation, nine participants infected with HTLV-1 were classified as probable TSP/HAM, and five individuals infected with HTLV-1 were classified as having definite TSP/HAM. We excluded subjects with neurological manifestations, inflammatory or infectious processes, other diseases that affect balance control, pregnancy, cognitive deficits, or people with disabilities infected with HTLV-1 who cannot maintain a quiet standing posture.

### 2.3. Static Balance-Control Evaluation

We used a force platform (model BIOMEC400, EMG System do Brazil, Ltda., São Paulo, Brazil) with spatially distributed load sensors in intervals of 50 cm^2^ connected to a computer using Biomec software (EMG System do Brazil, Ltda., Brazil) to start and end the recordings at a data acquisition rate of 100 Hz. All participants were in barefoot conditions during all body sway evaluations. We asked them to move from the force platform and remain in a quiet standing position with shoulders wide apart, arms at sides, and to gaze at a point one meter away. The duration of a single trial was 60 s. Each participant made three attempts with open and closed eyes with an interval of 1 min between the trials.

### 2.4. Data Analysis

The time series of the COP displacement were exported in text files that were processed in MATLAB routines (MATLAB R2020a, MathWorks, Natick, EUA). We detrended the time series of the COP displacement in the anteroposterior and mediolateral axes followed by a bidirectional second-order, low-pass Butterworth filtering at 10 Hz. We excluded the first 5 s of the time series, and 55 s of the recordings were analyzed. The time series were analyzed in three intervals: 0–10 s (T1 period), 10–45 s (T2 period), and 45–55 s (T3 period). The COP displacements were quantified by the following parameters:

(i) Structural parameters. To quantify the structural parameters of the COP displacement, a Sway Density Curve (SDC) was created, which quantified the number of consecutive samples per time unit inside a circle with a radius of 2.5 mm. The number of samples quantified at each time unit was divided by the acquisition rate of the platform. The SDC was filtered by a second-order Butterworth low-pass filter at 2.5 Hz. From the SDC, the structural parameters, MP, MD, and MT, were extracted. MP was the mean value of the peaks (in seconds); MD was the mean distance between successive peaks (in millimeters); MT was the mean interval between successive peaks (in seconds) [27]. 

(ii) Global parameters: global parameters were quantified from stabilometric measures and from the statokinesiogram. The MATLAB commands are used to calculate each global parameter, as shown in Table 1 [28].

### 2.5. Statistics

We averaged three trials for each test condition to proceed the statistical analyses. The Shapiro–Wilk test was used to evaluate the data distribution. One-way ANOVA followed by Tukey’s test or the Kruskal–Wallis test, followed by Dunn’s test was applied for parametric and nonparametric variables, respectively, to assess the effect of the group (probable and definite TSP/HAM groups, and the control group) on the displacement parameters of COP, considering the period (T1, T2, and T3) and experimental condition (open and closed eyes) as fixed factors. A confidence level of 5% was considered for all statistical procedures.

## 3. Results

### 3.1. Sample Features

The sample consisted of 29 participants grouped in the control group (n = 16, 45.8 ± 8.47 years) and participants infected with HTLV-1 (n = 13). After the neurological exam to classify the level of TSP/HAM ascertainment, we observed that eight participants infected with HTLV-1 were classified as probable TSP/HAM (55 ± 9.35 years), and five participants infected with HTLV-1 were classified as definite TSP/HAM (52.4 ± 8.6 years).

Considering the time since the diagnosis of HTLV-1 infection, participants with probable TSP/HAM had 11.7 ± 6.7 years and the group with definite TSP/HAM had 8.8 ± 3.9 years. For the demographic variables of age, weight, and height, no significant values were found between the studied groups (*p* < 0.05). Table 2 shows the features of all groups.

### 3.2. Static Balance-Control Evaluation

**Figure 1:**
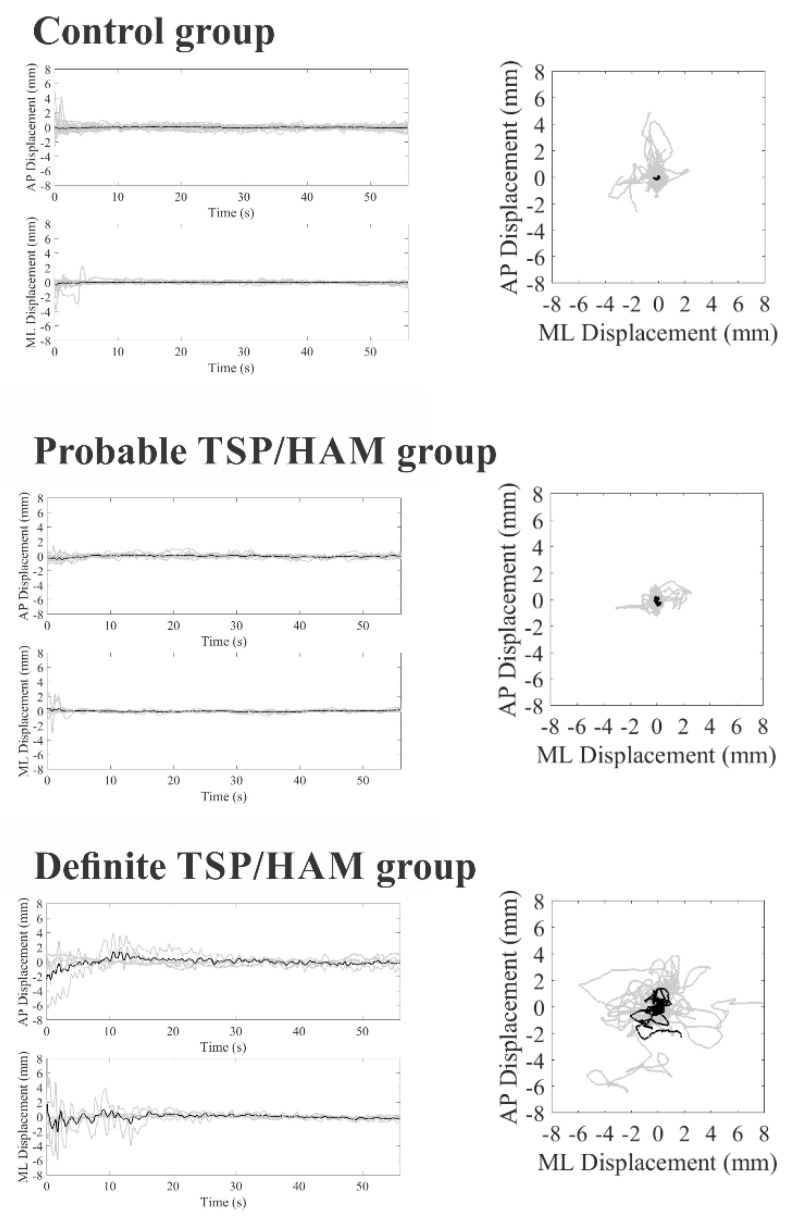
COP displacements in the anteroposterior (AP) and mediolateral (ML) axis as a function of the duration of the test (on left) and statokinesiograms (on right) of the control group, probable TSP/HAM group, and definite TSP/HAM group under the open-eye condition. Gray recordings represent individual waveforms; black recordings represent the grand average recordings.

**Figure 2:**
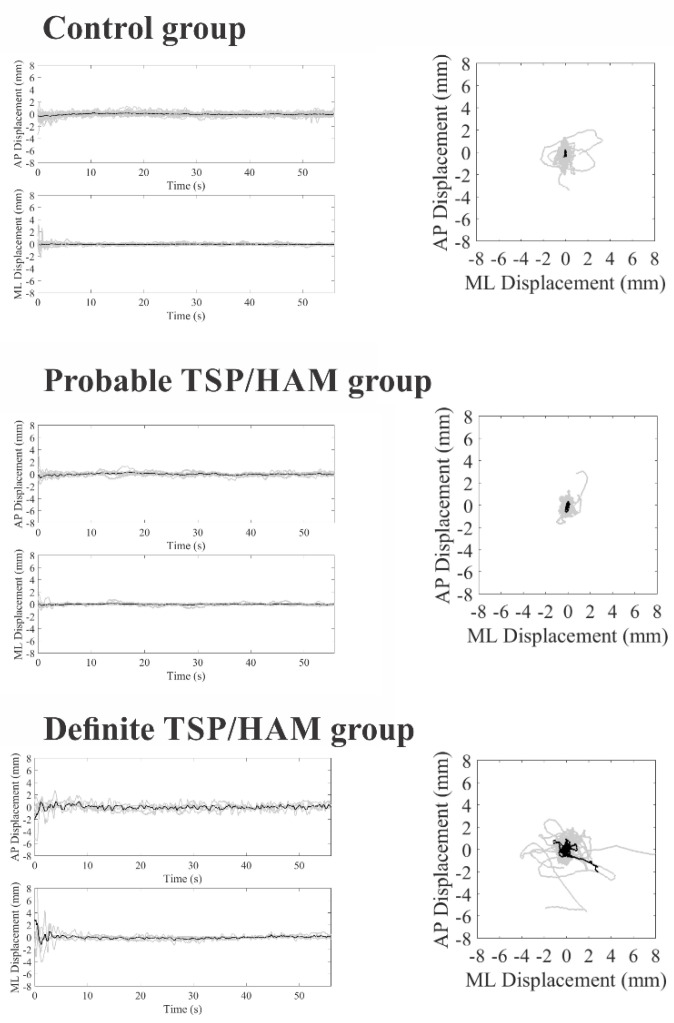
COP displacements in the anteroposterior (AP) and mediolateral (ML) axes as a function of the duration of the test (on left) and statokinesiograms (on right) of the control group, probable TSP/HAM group, and definite TSP/HAM group in the closed-eye condition. Gray recordings represent individual waveforms; black recordings represent grand average recordings.

Figure 1 and Figure 2 show stabilometric recordings on the anteroposterior and mediolateral axes and statokinesiograms for the control group, the probable TSP/HAM group, and the definite TSP/HAM group for the open- and closed-eyes conditions, respectively. 

### 3.3. Structural Parameters of the COP

Table 3 shows the mean and standard deviation of the MP, MT, and MD variables for all groups, and the static balance evaluation conditions in the three periods of the time series of the body sway recordings. 

In open-eye conditions, there was no significant effect of the group factor on the MT values for the three periods of the recordings (period T1: F [2, 26] = 0; *p* = 0.79; period T2: F [2, 26] = 0.99; *p* = 0.39; period T3: X² [2, 29] = 0.317; *p* = 0.853). Furthermore, there was a significant effect of the group factor on MD values during the period T1: (F [2, 26] = 4.14; *p* = 0.027), in which the definite TSP/HAM group had higher values compared to the control group (*p* < 0.05). No significant differences were found in the MD values in the other periods of the recording (period T2: F [2, 26] = 1.23; *p* = 0.31; period T3: F [2, 26] = 0.76; *p* = 0.48). We observed that the group factor had a significant influence on MP values during the three periods of the body sway recordings (period T1: X² [2, 29] = 8.117; *p* = 0.017; period T2: X² [2, 29] = 7.823; *p* = 0.02; period T3: X² [ 2, 29] = 9.796; *p* = 0.007) in which the control group obtained higher values than the definite TSP/HAM group (*p* < 0,05). Additionally, the probable TSP/HAM group obtained higher values than the definite TSP/HAM group (*p* < 0.05) in the first time interval only.

For the closed-eye conditions, the group factor had no significant influence on the MT and MD values in the periods T1, T2, and T3 (MT values. Period T1: F [2, 26] = 0.37; *p* = 0.69; period T2: F [2, 26] = 0.85; *p* = 0.44; period T3: X² [2, 29] = 0.448; *p* = 0.799; MD values. T1 period: F [2, 26] = 1.08; *p* = 0.35; period T2: F [2, 26] = 0.19; *p* = 0.82; period T3: F [2, 26] = 0.13; *p* = 0.88). However, there was a significant influence of the group factor on the MP values of the periods (T1: X² [2, 29] = 7.072; *p* = 0.029; T2: X² [2, 29] = 7.478; *p* = 0.024, and T3: X² [2,29] = 6.695; *p* = 0.035). During all periods, the control participants had higher values than the participants with definite TSP/HAM (*p* < 0.05), and the participants of the probable TSP/HAM group obtained values higher than the definite TSP/HAM group (*p* < 0.05) in the T1 and T2 intervals. There were no statistical differences in the other multiple comparisons.

### 3.4. Global Parameters of the COP: Time-Domain Parameters

Table 4 shows the mean and standard deviation of the global parameters in the time domain (RMSX, RMSY, area) under the open- and closed-eye conditions. Under the open-eye condition, there was a significant effect of the group factor on the amplitude of RMS on the mediolateral axes (RMSX) in the period T1: X² [2, 29] = 8.055; *p* = 0.018; T2 (F [2, 26] = 8.96; *p* = 0.001), and period T3 (X² [2, 29] = 7.136; *p* = 0.028). During all periods, the control group had significantly lower values than the definite TSP/HAM group (*p* < 0.05). In the T2 interval, we observed a statistical difference between the groups infected with HTLV-1, definite TSP/HAM, and probable TSP/HAM (*p* < 0.05), while all other multiple comparisons did not find a significant difference (*p* > 0.05). There was no significant influence of the group factor on the RMSY parameter in the first two periods of recording body movement (period T1: X² [2, 29] = 1.964; *p* = 0.375; period T2: X² [2, 29] = 4.984; *p* = 0.083); however, there was a significant difference in the T3 (period T3: X² [2, 29] = 6.027; *p* = 0.049). The control group showed significantly lower values than the definite TSP/HAM group (*p* < 0.05). The influence of the groups in the ellipse area of the statokinesiogram was not significant in the first period (T1 period: X² [2, 29] = 5.093; *p* = 0.078), but it was significant in the periods (T2: X² [2, 29] = 9.273; *p* = 0.01) and period (T3: X² [2, 29] = 8.036; *p* = 0.018). The definite TSP/HAM group had larger areas than the control group (*p* < 0.05) in significant periods.

In the closed-eye condition, there was a significant influence of the group factor on the RMSX values in the T1 period (F [2, 26] = 7.8; *p* = 0.002) and the period T3 (X² [2, 29] = 7.488; *p* = 0.024). In periods T1 and T3, the control participants had significantly lower values than the participants with defined TSP/HAM (*p* < 0.05). In the T1 period, the definite TSP/HAM group had a higher RMSX value than the probable TSP/HAM group ( *p* < 0.05). However, there was no significant influence in the T2 period (X² [2, 29] = 5.461; *p* = 0.065). For RMSY, we observed that the group had a significant influence on the RMSY values in the T1 periods T1 (X² [2, 29] = 7.136; *p* = 0.03) and T2 (X² [2, 29] = 7.807; *p* = 0.02); in both periods, participants in the control group had significantly lower RMSY values than participants in the definite group; in addition to this, in period T2, the definite TSP/HAM group had a higher RMSY value than the probable TSP/HAM group ( *p* < 0.05). However, there was no significant effect on the T3 interval T3 ( X² [2, 29] = 5.28; *p* = 0.073). The effect of the groups on the ellipse area of the statokinesiogram was significant in period T1 (X² [2, 29] = 7.765; *p* = 0.021), period T2 (X² [2, 29] = 7.332; *p* = 0.026), and period T3 ( X² [2, 29] = 6.403; *p* = 0.041), in which the definite TSP/HAM group presented a larger ellipse area than the controls (*p* < 0.05).

### 3.5. Global Parameters of the COP: Temporal Frequency Parameters

Table 5 shows the mean and standard deviation of the global temporal frequency parameters of the COP. In open-eye condition, there was no significant effect of the group factor on the median frequency on the mediolateral axes, MFX (period T1: X² [2, 29] = 3.729; *p* = 0.155; period T2: X² [2, 29] = 2.046; *p* = 0.359; period T3: X² [2, 29] = 2.793; *p* = 0.248) There no was a significant influence of the group factor on the median frequency on the anteroposterior axes (MFY) during period T1 (X² [ 2, 29] = 3,.4; *p* = 0.147) and period T2: X² [2, 29] = 5.549; *p* = 0.062) and period T3 (X² [2, 29] = 3.747; *p* =0.154).

For the closed-eye conditions, there was no significant influence of the group factor on the median frequency on the mediolateral axes during all periods (T1 period: X² [2, 29] = 2.104; *p* = 0.349; period T2: X² [2, 29] = 0.592; *p* = 0.744; period T3: X² [2, 29] = 3.432; *p* = 0.18). There was no significant influence of the group factor on the median frequency on the anteroposterior axes during the entire period (T1 period: X² [2, 29] = 0.148; *p* = 0.929; period T2: X² [2, 29] = 1.43; *p* = 0.489; period T3: X² [2, 29] = 1.368; *p* = 0.505).

### 3.6. Summary of the Results

**Figure 3:**
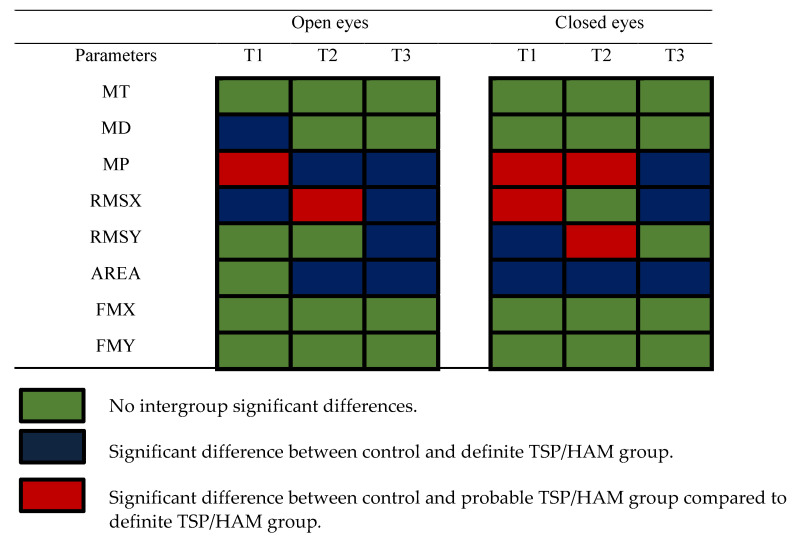
Summary of the significance of the results from the definite TSP/HAM group.

We evaluated the balance control using eight parameters of the open-eye condition and eight parameters of the closed-eye condition. Only the definite TSP/HAM group had significant differences compared to the controls at different recording periods. Figure 3 shows a graphical summary of the results, and we can observe that in the open-eye condition, 3/8 parameters were altered in periods T1 and T2, while 4/8 parameters were altered in period T3. In the closed-eye condition, we observed 4/8 altered parameters in period T1, while 3/8 parameters were altered in period T2 and T3.

## 4. Discussion

Our main findings showed that participants infected with HTLV-1 with definite TSP/HAM had a significant loss of balance control, while HTLV-1-infected participants with probable had a significant loss of balance in some parameters compared to the definite group.

Our results agree with previous findings that subjects infected with HTLV-1 presenting TSP/HAM have balance-control impairments [16,22,23,29]. The present novelty is that among the subjects infected with HTLV-1 with TSP/HAM, it was possible to distinguish two groups of patients according to the level of TSP/HAM ascertainment that differed in balance-control functionality. While the participants of the probable group in some analyzed variables showed a significant loss of balance control in relation to the definite HTLV group, when analyzed in relation to the control group, we did not observe significant differences. Subjects with definite TSP/HAM had a series of losses in the static balance control. Previously, it was reported [22] that subjects infected with HTLV-1 without TSP/HAM could present an intermediate balance between the controls and patients with symptoms of TSP/HAM. In the present study, we observed that the probable TSP/HAM group and control group had similar balance control results. For some parameters, there was a significant difference between the probable and definite TSP/HAM groups, indicating that patients with probable TSP/HAM could be, in some intermediate place, in a continuum of symptoms that lead from the controls to the patients infected with HTLV-1 with definite TSP/HAM.

Previously, our group reported a comparison of posturographic parameters from the controls, asymptomatic HTLV-1-infected patients, and symptomatic HTLV-1-infected patients, and reported that patients with TSP/HAM had a loss of balance control compared to healthy participants [23]. We reported that the asymptomatic patients had an intermediate balance-control profile between the control group and patients with TSP/HAM, but most of the differences between the asymptomatic patients and the controls were non-significant. Here, in the present investigation, we observed a similar relationship between the controls and patients with probable TSP/HAM—although there were non-significant differences between them, the patients with probable TSP/HAM usually had intermediate values between the controls and patients with definite TSP/HAM.

The interpretation of the 16 parameters for the evaluation of the COP displacement enables one to describe the features of the compensation mechanism to keep the posture in the definite TSP/HAM group. Changes in the pattern of body oscillation occurred more in amplitude than in frequency. The amplitude of the oscillations is represented by MP, MD, RMS, and area parameters; the sway frequency is represented by MT and the median frequency. As shown in Figure 3, the parameters that measured the frequency of the sway had more non-significant differences compared to the controls than the parameters that measured the amplitude of the sway.

Patients with TSP/HAM had a higher proviral load than asymptomatic patients, thus, showing that this inflammatory condition would influence the onset of the myelopathy [30,31]. High levels of proinflammatory cytokines and chemokines are found in patients with TSP/HAM and are probably associated with TSP/HAM pathogenesis [32]. No differences in proviral load and cytokines/chemokines have been reported. The complete mechanism to the neurological damage in the TSP/HAM is still unclear, but is probably related to direct toxicity, autoimmunity, and bystander damage (for more details, see [33]). No previous investigations reporting balance-control losses in TSP/HAM patients explored the association of these biomarkers and the extension of balance loss [31].

A limitation of our findings was the number of patients with TSP/HAM (n = 13), and that they were split into two groups (probable and definite TSP/HAM). Our patients with TSP/HAM also participated from a previous study [23], whereby all of them were included in the group with TSP/HAM; in the current study, we observed that significant differences regarding the patients compared to the controls occurred mainly because the losses in the present study were presented by patients who were identified as having definite TSP/HAM. Future investigations can increase the number of patients with different levels of TSP/HAM ascertainment. In addition to previous studies, the present results display the use of the balance-control evaluation to monitor the neurological damage progression in patients with TSP/HAM.

The present investigation added new information on the pathophysiology of balance loss in HTLV-1 patients with TSP/HAM. The severity of balance control depended on the level of TSP/HAM ascertainment, the viewing test condition, and the moment of recording body sway. Knowledge of these results may be important to guide a better functional evaluation of these patients for treatment and rehabilitation.

## Figures and Tables

**Table 1:** MATLAB commands for global parameters.

Parameter	Description	MATLAB Command
Ellipse area	Area including 95% of the statokinesiogram.	$\left[vec,val\right]=eig\left(cov\left(COP_{AP},COP_{ML}\right)\right)$ $area=pi*prod\left(2.4478\times \sqrt{svd\left(val\right)}\right)$
RMS	Magnitude of the COP displacement.	$RMS_{AP}=\sqrt{\overset{n}{\underset{i=1}{\sum }}{⬚\left(COP_{AP_{i}}\right)}^{2}}$ $RMS_{ML}=\sqrt{\overset{n}{\underset{i=1}{\sum }}{⬚\left(COP_{ML_{i}}\right)}^{2}}$
Median frequency	The median frequency is the frequency that limits a frequency band that contributes 50% of the power spectrum.	$nfft=round(\frac{length\left(COP\right)}{2})$ $\left[p,f\right]=pwelch\left(COP,\left[⬚\right],\left[⬚\right]nfft,\;Fs\right)$ $\left[m,peak\right]=max\left(p\right)$ $area=cumtrapz\left(f,p\right)$ $find50=find\left(area\geq 0.5\times area\left(end\right)\right)$ $medianFreq_{AP}=f\left(find50\left(1\right)\right)$

COP: center of pressure displacement; AP: anteroposterior; ML: mediolateral; Fs = sampling frequency.

**Table 2:** Characteristics of the groups.

	Control	Probable TSP/HAM	Definite TSP/HAM
Sex	8 F, 8 M	2 M; 6 F	1 M; 4 F
Age (years)	45.8 ± 8.47	55 ± 9.3	52.4 ± 8.6
Height (meter)	1.66 ± 0.7	1.60 ± 0.1	1.57 ± 0.2
Weight (kilogram)	71.0 ± 13.2	72.3 ± 14.5	62.6 ± 6.5
Duration of the disease (year)	NA	11.75 ± 6.7	8.8 ± 3.9
Use of orthosis	NA	12.5%	40%
Rehabilitation	NA	50%	100%
Weakness or fatigue in the legs	NA	75%	100%
Sphincter disturbances	NA	62.5%	100%
Sensitive disturbances	NA	75%	80%
Low back pain	NA	75%	80%
Difficulty climbing stairs	NA	62.5%	100%
Difficulty walking long distances	NA	62.5%	100%
History of falls	NA	25%	60%

NA: not applicable; F: female; M: male.

**Table 3:** The mean (standard deviation) of the structural variables of the displacement of the COP.

	Open Eyes			Closed Eyes		
Variable	MP	MT	MD	MP	MT	MD
*Period T1*						
Control	12.14 (7.35)	0.69 (0.07)	0.11 (0.02)	11.29 (5.76)	0.68 (0.06)	0.11 (0.02)
Probable TSP/HAM	9.44 (6.15)	0.68 (0.10)	0.09 (0.03)	10.98 (3.61)	0.68 (0.70)	0.10 (0.04)
Definite TSP/HAM	3.190 ** **(2.84)**	0.656 (0.12)	0.140 * **(0.02)**	3.97 ** **(4.32)**	0.653 (0.10)	0.127 (0.02)
*Period T2*						
Control	18.5 (7.4)	0.69 (0.05)	0.11 (0.02)	16.39 (7.08)	0.66 (0.05)	0.11 (0.02)
Probable TSP/HAM	13.97 (7.4)	0.68 (0.09)	0.09 (0.03)	14.69 (5.74)	0.68 (0.06)	0.10 (0.04)
Definite TSP/HAM	7.0 * **(6.97)**	0.64 (0.04)	0.11 (0.02)	6.18 ** **(5.76)**	0.64 (0.05)	0.11 (0.02)
*Period T3*						
Control	19.95 (8.45)	0.67 (0.07)	0.11 (0.02)	16.5 (7.22)	0.67 (0.09)	0.11 (0.02)
Probable TSP/HAM	13.93 (7.1)	0.67 (0.11)	0.09 (0.03)	14.45 (6.0)	0.69 (0.09)	0.10 (0.05)
Definite TSP/HAM	7.04 * **(5.95)**	0.66 (0.03)	0.11 (0.02)	6.66 * **(6.43)**	0.64 (0.06)	0.11 (0.02)

* Significant difference compared to the control group. ** Significant difference compared to the control group and probable TSP/HAM.

**Table 4:** Mean (standard deviation) of the global parameters measured in the time domain.

	Control Group					
	Open Eyes			Closed Eyes		
Parameter	T1	T2	T3	T1	T2	T3
RMSX	0.61 (0.25)	0.32 (0.12)	0.19 (0.09)	0.63 (0.31)	0.32 (0.12)	0.18 (0.06)
RMSY	0.96 (0.47)	0.5 (0.19)	0.39 (0.15)	0.73 (0.36)	0.49 (0.15)	0.43 (0.17)
Area	3.9 (5.06)	1.5 (0.9)	0.77 (0.77)	3.11 (5.1)	1.54 (0.65)	0.88 (0.55)
	**Probable TSP/HAM group**					
	**Open eyes**			**Closed eyes**		
**Parameter**	**T1**	**T2**	**T3**	**T1**	**T2**	**T3**
RMSX	0.97 (0.58)	0.52 (0.25)	0.30 (0.16)	0.82 (0.31)	0.42 (0.18)	0.27 (0.13)
RMSY	1.3 (0.91)	0.67 (0.4)	0.52 (0.32)	1.11 (0.8)	0.55 (0.36)	0.5 (0.21)
Area	4.31 (7.53)	3.06 (2.14)	1.02 (0.5)	3.05 (1.73)	2.7 (3.0)	1.22 (0.7)
	**Definite TSP/HAM group**					
	**Open eyes**			**Closed eyes**		
**Parameter**	**T1**	**T2**	**T3**	**T1**	**T2**	**T3**
RMSX	2.32 * **(1.46)**	0.94 ** **(0.61)**	0.46 * **(0.19)**	1.97 ** **(1.54)**	0.73 * **(0.5)**	0.45 * **(0.27)**
RMSY	1.66 (1.4)	0.92 * **(0.43)**	0.7 * **(0.25)**	1.49 * **(0.68)**	0.98 ** **(0.33)**	0.78 * **(0.3)**
Area	57.67 (59.94)	12.26 * **(11.22)**	3.12 ** **(1.6)**	44.3 * **(52.5)**	7.85 * **(4.25)**	4.2 * **(2.8)**

RMSX: RMS amplitude on the ML axes; RMSY: RMS amplitude on the AP axes; AP: anteroposterior; ML: mediolateral. * Significant difference compared to the control group. ** Significant difference compared to the control group and probable TSP/HAM.

**Table 5:** Mean (standard deviation) of the global parameters measured in the frequency domain.

	Open Eyes		Closed Eyes	
Parameter	MFX	MFY	MFX	MFY
*Period T1*				
Control	0.43 (0.05)	0.42 (0.04)	0.42 (0.08)	0.45 (0.09)
Probable TSP/HAM	0.41 (0.02)	0.41 (0.02)	0.41 (0.02)	0.44 (0.07)
Definite TSP/HAM	0.46 (0.06)	0.48 (0.11)	0.45 (0.08)	0.46 (0.09)
*Period T2*				
Control	0.15 (0.06)	0.13 (0.02)	0.17 (0.08)	0.17 (0.06)
Probable TSP/HAM	0.15 (0.09)	0.14 (0.02)	0.12 (0.14)	0.2 (0.08)
Definite TSP/HAM	0.175 (0.06)	0.24 (0.1)	0.18 (0.15)	0.25 (0.17)
*Period T3*				
Control	1.26 (1.7)	0.42 (0.05)	1.11 (1.6)	0.41 (0.1)
Probable TSP/HAM	0.84 (1.22)	0.4 (0.08)	0.41 (0.04)	0.42 (0.05)
Definite TSP/HAM	0.97 (1.17)	0.5 (0.13)	0.54 (0.14)	0.52 (0.16)

MFX: median frequency on the ML axes; MFY: median frequency on the AP; peak frequency; AP: anteroposterior; ML: mediolateral.

## Data Availability

The data that support the findings of this study are available from the corresponding author upon reasonable request.
